# Ferritic Alloys with Extreme Creep Resistance via Coherent Hierarchical Precipitates

**DOI:** 10.1038/srep16327

**Published:** 2015-11-09

**Authors:** Gian Song, Zhiqian Sun, Lin Li, Xiandong Xu, Michael Rawlings, Christian H. Liebscher, Bjørn Clausen, Jonathan Poplawsky, Donovan N. Leonard, Shenyan Huang, Zhenke Teng, Chain T. Liu, Mark D. Asta, Yanfei Gao, David C. Dunand, Gautam Ghosh, Mingwei Chen, Morris E. Fine, Peter K. Liaw

**Affiliations:** 1 Department of Materials Science and Engineering, The University of Tennessee, Knoxville, TN, 37996-2200; 2 WPI Advanced Institute for Materials Research, Tohoku University, Sendai 980-8577, Japan; 3 Department of Materials Science and Engineering, Northwestern University, Evanston, IL 60208-3108; 4 Department of Materials Science and Engineering, University of California, Berkeley, CA 94720; 5 Lujan Center, Los Alamos National Laboratory, Los Alamos, NM 87545, USA; 6 Center for Nano phase Materials Sciences, Oak Ridge National Laboratory, Oak Ridge, TN 37831, USA; 7 Center for Advanced Structural Materials, Department of Mechanical and Biomedical Engineering, City University of Hong Kong, Kowloon, Hong Kong

## Abstract

There have been numerous efforts to develop creep-resistant materials strengthened by incoherent particles at high temperatures and stresses in response to future energy needs for steam turbines in thermal-power plants. However, the microstructural instability of the incoherent-particle-strengthened ferritic steels limits their application to temperatures below 900 K. Here, we report a novel ferritic alloy with the excellent creep resistance enhanced by coherent hierarchical precipitates, using the integrated experimental (transmission-electron microscopy/scanning-transmission-electron microscopy, *in-situ* neutron diffraction, and atom-probe tomography) and theoretical (crystal-plasticity finite-element modeling) approaches. This alloy is strengthened by nano-scaled L2_1_-Ni_2_TiAl (Heusler phase)-based precipitates, which themselves contain coherent nano-scaled B2 zones. These coherent hierarchical precipitates are uniformly distributed within the Fe matrix. Our hierarchical structure material exhibits the superior creep resistance at 973 K in terms of the minimal creep rate, which is four orders of magnitude lower than that of conventional ferritic steels. These results provide a new alloy-design strategy using the novel concept of hierarchical precipitates and the fundamental science for developing creep-resistant ferritic alloys. The present research will broaden the applications of ferritic alloys to higher temperatures.

Creep resistance is one of the most important mechanical properties for elevated-temperature structural applications, such as components for steam turbines in thermal-power plants^1^,^2^,^3^,^4^,^5^,^6^. Ferritic steels strengthened by nano-scaled particles have been employed for steam turbines in thermal-power plants, due to their good thermal conductivity, low thermal expansion, and cost efficiency, as compared to austenitic steels and Ni-based superalloys^7^,^8^,^9^,^10^,^11^,^12^,^13^,^14^. However, the coarsening behavior of the incoherent strengthening carbides and the microstructural instability (e.g., Z-phase formation) limit the application of ferritic steels to temperatures below 873 K^15^,^16^,^17^,^18^,^19^. Currently, ultra-supercritical (USC) steam turbines require an increased steam temperature and pressure (up to 1,033 K and 35 MPa, respectively)^20^,^21^. Advanced ferritic steels with the increased permissible operating temperatures (up to 923 K) have been developed by conventional alloy-design strategies, such as solid-solution strengthening and nano-scaled carbide- or nitride-type precipitate reinforcement^10^,^22^,^23^, but the achieved creep properties were not satisfactory to meet the USC requirement.

The NiAl-strengthened ferritic alloys have been investigated because of their excellent creep and oxidation resistance^24^,^25^,^26^,^27^,^28^. The microstructure of NiAl-ferritic alloys is analogous to that of the γ/γ′ Ni-based alloys, consisting of a disordered body-centered-cubic (BCC) α-Fe matrix and an ordered B2-structured NiAl phase^29^, providing the possibility of achieving a similar strengthening effect in γ/γ′ Ni-based superalloys at elevated temperatures^30^. However, the key issue for the NiAl-strengthened ferritic alloys is their lack of sufficient creep resistance at 973 K for steam-turbine applications^24^. In particular, the creep resistance at low rupture stresses is comparable, but as the rupture stress increases, the creep resistance becomes inferior to other Fe-based candidates^24^.

In the present research, a fundamental study and development strategy of novel ferritic alloys consisting of either single-phase Ni_2_TiAl precipitates or hierarchical NiAl/Ni_2_TiAl precipitates are reported. Systematic investigations have been conducted, using the integrated experimental [scanning/transmission-electron microscopy (S/TEM), *in-situ* neutron diffraction (ND), and atom-probe tomography (APT)] and theoretical (crystal-plasticity finite-element modeling) approaches. The creep resistance of the investigated alloys at 973 K is significantly improved, as compared to the NiAl-strengthened materials and other Fe-based candidates. Moreover, the alloy with hierarchical precipitates shows much better creep resistance than the single Ni_2_TiAl-precipitate-strengthened alloy. The main objectives of the present study are to understand (1) the effects of precipitate structures on creep behavior and (2) the crucial factors contributing to the superior creep resistance of novel ferritic alloys with hierarchical precipitates. The in-depth understanding of the relationship between microstructures and creep properties will provide the necessary insight for the design and optimization of creep-resistant ferritic alloys and for wide and practical applications in advanced steam-turbine systems.

The prototype precipitation-strengthened ferritic alloy with a composition of Fe-6.5Al-10Cr-10Ni-3.4Mo-0.25Zr-0.005B weight percent (wt%) (denoted as FBB8 in this study) consists of coherent B2-NiAl precipitates in the BCC Fe matrix (Fig. 1a) with a volume fraction (vol%) of 16 ~ 18% and an average diameter of 130 nm after the heat treatment (a solution treatment at 1,473 K for 0.5 hours, followed by aging at 973 K for 100 hours, see the Methods Section: Materials)^31^. The morphology of the coherent precipitate is a function of misfit strains and interfacial energies, and the spherical shape of the NiAl precipitates indicates the dominance of the interfacial energy and low misfit strain between the matrix and precipitate^32^, which is supported by our previous ND study (a lattice mismatch of 0.01% at 973 K)^33^. The FBB8 possesses a creep resistance at 973 K, comparable to well-known conventional ferritic steels (P92, P122, T91, T122, and 12Cr)^17^,^34^,^35^,^36^,^37^ (Fig. 2a,b) (See Supplementary Table S1 for the detailed compositions of the compared ferritic steels). The permissible operating temperatures of the compared commercial ferritic steels are generally limited to 900 K^22^, which implies that the creep resistance of FBB8 is not sufficient for industrial applications at temperatures higher than 900 K.

Our approach to improve the creep resistance of the ferritic alloys is to introduce a new type of two-phase precipitates with intrinsically-superior high-temperature properties, while retaining the interfacial coherency between the matrix and precipitate^38^. From the available literature, the L2_1_ (Heusler phase) phase has an excellent creep resistance in the temperature range from 1,026 to 1,273 K^39^, and a similar lattice structure/constant for the small lattice mismatch with the BCC Fe structure^40^,^41^, which satisfies the requirements of our microstructural design. Another strategy for the further improvement of the creep resistance is to establish a hierarchical precipitate comprising a network of B2-NiAl and L2_1_-Ni_2_TiAl phases. The hierarchical structure is characterized by the relative chemical ordering, spatial dimensions of the phases, and their spatial distribution^42^. Specifically, the Fe-based matrix features the largest length scale, whereas the primary precipitates are characteristic of a smaller length scale. The primary precipitate is further divided by the sub-structure at the lowest length scale. The matrix has a chemically-disordered structure (disordered body-centered-cubic), while the constitutive phases in the primary precipitate have chemically ordered structures (B2 and L2_1_ structures are different from each other in terms of the sublattice occupancy). The hierarchical structure has been developed and reported in ribbon samples of Fe–Al–Cr–Ni–Ti alloys, and been used to describe such a microstructure of the two-phase precipitate in the ferritic alloys^42^,^43^. In order to design novel hierarchical NiAl/Ni_2_TiAl precipitation-strengthened ferritic alloys and single Ni_2_TiAl precipitation-strengthened ferritic alloys (denoted as HPSFA and SPSFA, respectively, in this study), 2 or 4 wt% Ti elements were added to the prototype FBB8 alloy, respectively.

## Results

### Transmission-electron microscopy microstructural characterization

**Figure 1:**
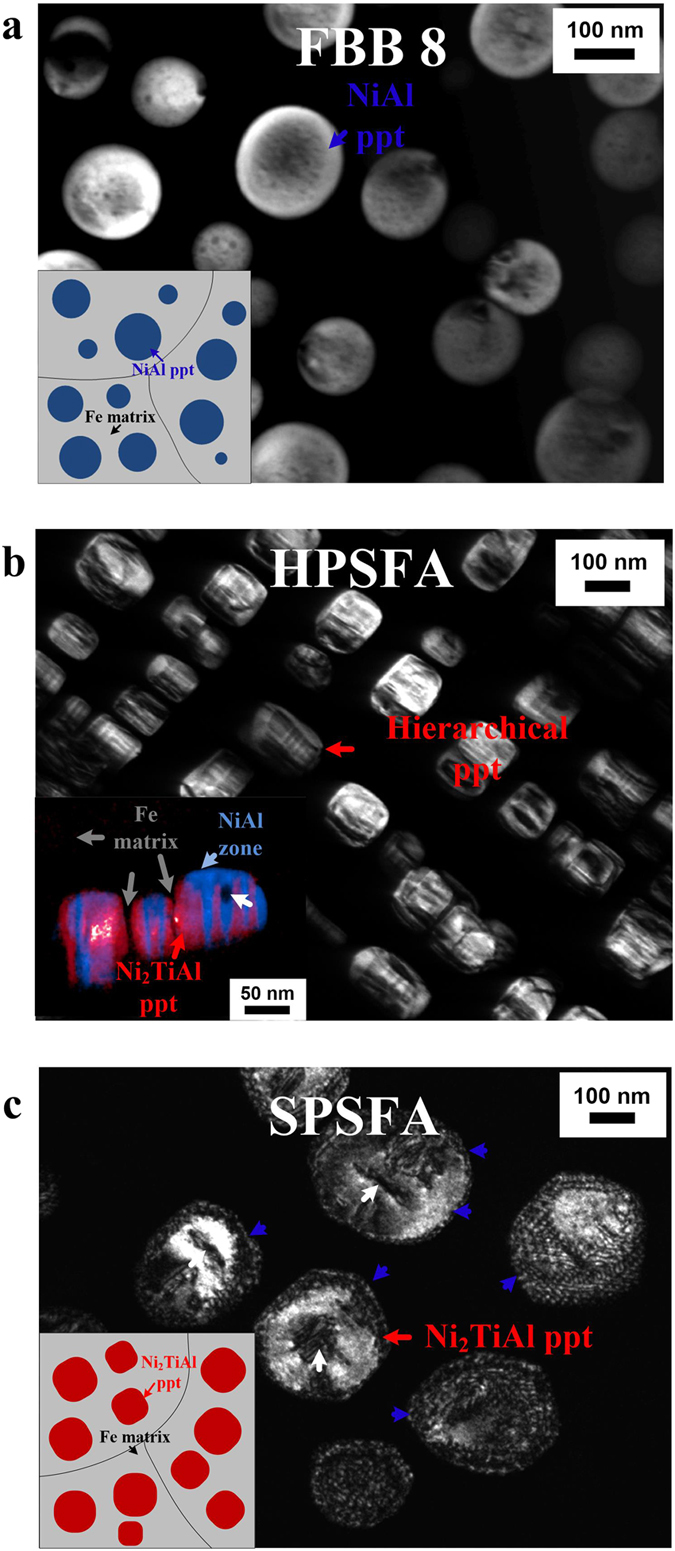
DF-TEM characterization of the precipitates-strengthened ferritic alloys. Dark-field (DF) transmission-electron-microscopy (TEM) images showing the microstructures of (**a**) FBB8. (**b**) HPSFA. and (**c**) SPSFA, and a schematic in each inset of (**a**–**c**) illustrating the microstructures of the corresponding alloys. The DF image of HPSFA in an inset of (**b**) showing a colored DF image combined with two DF images taken along the [101] zone using <111> (red) and <020> (blue) superlattice reflections (ppt stands for precipitate). White arrows in (**b**-**c**) are Fe inclusion.

A dark-field TEM image of the HPSFA microstructure after the heat-treatment process (a solution treatment at 1,473 K for 0.5 hours, followed by aging at 973 K for 100 hours) is shown in Fig. 1b. The HPSFA contains an average volume fraction of 16.3 ± 2.3% cuboidal precipitates with an average width of 98 ± 23 nm and length of 111 ± 27 nm embedded in the Fe matrix. A Fe-15.4Al-12.1Cr-1.0Mo-16.0Ni-4.3Ti (in atomic percent) ribbon sample was studied, using TEM, to characterize the detailed microstructures of the two-phase precipitates consisting of B2-NiAl and L2_1_-Ni_2_TiAl phases^43^. A dark-field (DF)-TEM technique was used on the same region employing different reflections unique to the superlattice-ordered precipitate phases (Supplementary Figure S1). The inset of Fig. 1b shows a false color DF-TEM image comprised of two DF-TEM images acquired along the [101] zone using the <111> and <020> superlattice reflections, which clearly shows the formation of narrow B2-type zones inside the parent L2_1_-type precipitate. Besides the B2 and L2_1_ phases in the precipitate, dark contrast zones are often observed inside the precipitates of HPSFA and SPSFA, as indicated by white arrows in Fig. 1b,c, and are identified as an Fe inclusion by the energy-dispersive X-ray spectroscopy (EDS) analysis in the TEM. It is worth noting that the B2/L2_1_ and matrix/precipitate interfaces are devoid of misfit dislocations (the inset of Fig. 1b). The cuboidal shape without the interfacial misfit dislocations implies that the precipitate/matrix interface is coherent with a higher misfit strain, as compared to FBB8. Similarly, the flat B2/L2_1_ interface without misfit dislocations also indicates the coherency of the interface with a high misfit strain, which is in contrast to the two-phase NiAl-Ni_2_TiAl alloy (with a high density of interfacial dislocations)^44^. However, the SPSFA with 4 wt% Ti contains single-phase L2_1_-type precipitates and with an average size of 220 ± 46 nm and volume fraction of 22.3 ± 2.2%, which are greater than those of HPSFA, as shown in Fig. 1c. Another striking difference between HPSFA and SPSFA is the morphology of the precipitates. The precipitates in SPSFA are elliptical in shape and are decorated with interfacial dislocations, indicative of a higher lattice mismatch between the precipitate and matrix than HPSFA, as denoted by blue arrows in Fig. 1c. It is known that the formation of the interfacial dislocations partially releases the misfit strain at the matrix/precipitate interface^45^, which can lead to a transition to an elliptical shape of the precipitate. Therefore, the L2_1_-type precipitates of SPSFA are semi-coherent with the reduced misfit strains at the matrix/precipitate interface, relative to that of HPSFA.

### Creep resistance at 973 K

**Figure 2:**
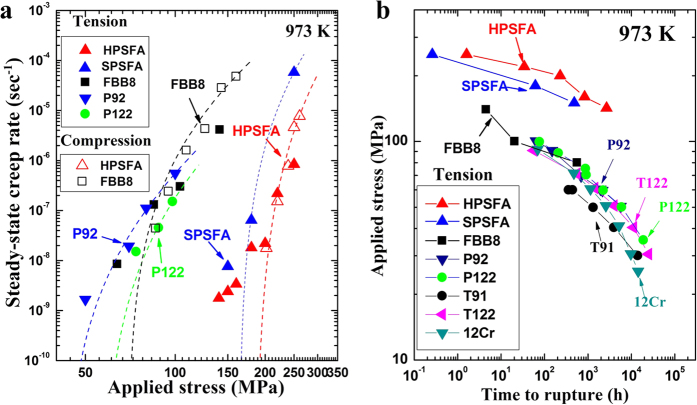
Comparison of the creep resistance of the precipitates-strengthened ferritic alloys. (**a**) A plot of the steady-state creep rate versus applied stress for HPSFA, SPSFA, FBB8, and commercial ferritic steels (P92 and P122)^17^ from compression and tension creep tests at 973 K, and best-fit curves obtained from a linear least-squares regression of 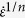 vs. 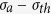 with n = 4 are also indicated. (**b**) A plot of the applied stress versus time to rupture at 973 K on the log-log basis for HPSFA, SPSFA, FBB8, and commercial ferritic steels (P92, P122, T91, T122, and 12Cr)^17^,^34^,^35^,^36^,^37^.

The creep resistance (the steady-state creep rate and time to rupture as a function of the applied stress) of HPSFA at 973 K was compared with FBB8, SPSFA, and conventional ferritic steels of P92, P122, T91, T122, and 12Cr in Fig. 2a,b^17^,^34^,^35^,^36^,^37^. Tension and compression creep tests were conducted (see the Methods Section: Tension and Creep Experiments at Elevated Temperatures), which cover a wide range of strain rates from 10^−4^ to 10^−9^ s^−1^, and the creep rates derived from both tension and compression tests are comparable to assure the reliability of the creep experiments in Fig. 2a. The steady-state creep rate of HPSFA at 973 K is significantly reduced by more than four orders of magnitude at corresponding stresses (Fig. 2a), compared to FBB8 and conventional ferritic steels^17^,^37^. The apparent stress exponent (n_app_) is measured, using the Power law,


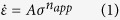


where 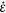 is the steady-state strain rate, A is a constant, and 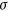 is the applied stress. The estimated value of n_app_ is higher than 4, observed in single-phase alloys^45^. In precipitate-strengthened alloys, the strain rate can be expressed, using the modified version of the creep equation^46^. In precipitate-strengthened alloys, the strain rate can be expressed, using the modified version of the creep equation^47^,


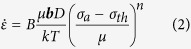


where *B* is a constant, μ is the shear modulus of the matrix, ***b*** is the Burger’s vector in the matrix, *D* is the effective diffusivity of a controlled element in the matrix, 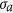 is the applied stress, *σ*_*th*_ is the threshold stress, and *n* is the stress exponent of the matrix. The threshold stress at strain rates above 1 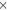 10^−8^ s^−1^, estimated by a linear least-squares regression of 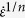 vs. *σ_a_−σ*_*th*_ with n = 4, is about 186 MPa. This value is more than two times higher than that of FBB8 (69 MPa)^37^. Moreover, the time to rupture at 142 MPa (2,675 hours) is more than two orders of magnitude greater than FBB8 at 140 MPa (about 4.5 hours) (Fig. 2b). The creep curves of HPSFA at 973 K and 160 MPa (Supplementary Figure S3) exhibit a prolonged-secondary region, where the minimum creep rate remains constant, as the creep time increases, in the studied stress range (140 ~ 240 MPa), while those of FBB8 show a dominant extended-tertiary region. The prolonged secondary creep region in HPSFA gives a significantly-improved creep resistance than that in FBB8. In addition, HPSFA shows much better creep resistance than SPSFA with a higher volume fraction of the precipitates (Fig. 2a,b).

### *In-situ* neutron-diffraction experiments and crystal-plasticity finite-element modeling at 973 K

**Figure 3:**
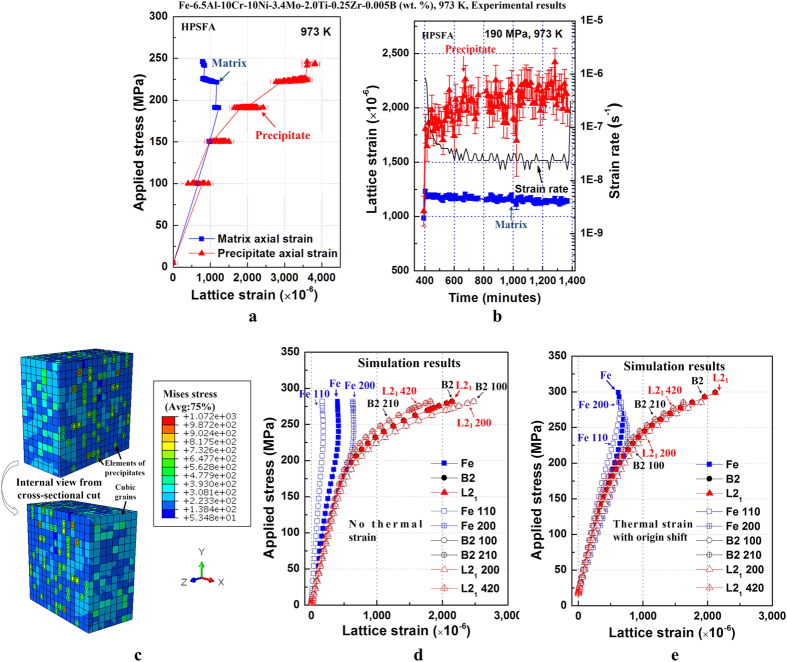
Lattice-strain evolution in HPSFA obtained from the *in-situ* creep ND experiments and CPFEM. (**a**) Average phase strains along the axial direction at 973 K as a function of applied stress during the entire *in-situ* creep experiments on HPSFA. (**b**) Average phase-strain evolutions of Fe and L2_1_ phases in the axial direction during *in-situ* creep deformation at 190 MPa and 973 K. (**c**) Schematic illustration of a 15 × 15 × 15 elements cubic model, employed in the simulations. The strain evolution of the Rietveld average and (hkl) plane lattices at 973 K under a uniaxial compressive stress, obtained using finite-element crystal-plasticity simulations (**d**) without and (**e**) with thermal residual stresses, respectively. Note that the (hkl) planes of the B2 and BCC Fe phases correspond to (2 h2 k2l) planes of the L2_1_ phase, since a L2_1_ unit cell contains eight unit cells of the B2 structure^39^. [Closed symbols: Rietveld average strains, Open symbols: (hkl) plane strains].

The step-loading tension creep tests on HPSFA, using *in-situ* ND, were carried out to understand the micro-deformation mechanisms at 973 K. Figure 3a exhibits the phase-average elastic lattice strain along the axial direction, determined from the Rietveld full pattern refinements^48^, as a function of the applied stress at 973 K, whereas more detailed *in-situ* ND results are shown in the in the Supplementary Information Section: ND Experiment. The slope of the axial precipitate lattice strain declines during loading to 190 and 220 MPa, relative to the linear slope during loading to 150 MPa, as shown in Fig. 3a. At the same time, the slope for the matrix becomes steeper, which indicates a load transfer from the matrix to the precipitate^49^. Moreover, during the *in-situ* ND creep testing at 190 MPa, a gradual increase in the precipitate lattice strain is observed, while the matrix lattice strain remains nearly constant (Fig. 3b), which indicates the load transfer from the matrix to the precipitate. The lattice strain versus stress relationship (Fig. 3a) provides key features in understanding the governing deformation mechanisms. Qualitatively, the splitting of lattice strains (Fig. 3a) indicates load sharing between the hard and soft material phases^49^. To investigate this load transfer further, a microstructure-based finite-element crystal-plasticity model was employed^50^. The constitutive law is discussed in the Supplementary Information Section: Finite-Element Crystal-Plasticity Model. The geometric setup of the model is shown in Fig. 3c. The grains are randomly oriented, while the precipitates have the same orientations as the matrix. A subset of <hkl> grains/phases are selected, with orientations parallel to the diffraction vector: The average Rietveld strains of the <hkl> subsets of all three phases were also taken and plotted in the same diagram to show agreement with the experimental results (Fig. 3d). The actual calculations use a tolerance of ±5° in angle, and 1 ~ 2% of all the grains were chosen to ensure a statistically-meaningful number^50^. A uniaxial compressive stress of 260 MPa was applied on the top surface of the cubic model at 973 K. Figure 3d shows that the matrix yields at around 200 MPa, and the matrix lattice strain starts to decrease. While the matrix yields, the L2_1_ and B2 precipitates absorb the applied stress and continue increasing their lattice strains, as the applied stress increases. These predictions (Fig. 3d) are in good agreement with the experimental results (Fig. 3a). This trend implies that the Fe matrix has reached its yield stress, while the two precipitates remains elastic at this stress level. In Fig. 3d, two <hkl> peaks were randomly chosen for each phase to be plotted in order to demonstrate the lattice-strain differences under the same loading conditions during the elastic-plastic behavior. The average lattice-strain values of <hkl> peaks were also taken and plotted in the figure for each phase to demonstrate the agreement of the simulation results with the experimental data (Fig. 3a,d). From this observation, it can be inferred that during loading to 190 MPa, the matrix starts to plastically deform, whereas the precipitate elastically deforms, and the load arising from the elastic/plastic misfit between the matrix and the precipitate is transferred to the precipitate that continues to carry strains, as experimentally verified in Fig. 3a. Similarly, as the creep time increases, the load from the creeping matrix is believed to be transferred to the elastically-deforming precipitate with increasing strains during creep at 190 MPa (Fig. 3b). The consideration of the initial thermal residual stress demonstrates the same trend in Fig. 3e.

## Discussion

Microstructural parameters, such as the volume fractions and the compositions of the constitutive phases (B2-NiAl, L2_1_-Ni_2_TiAl, and Fe phases) are closely related to the alloy chemistry^51^,^52^. The volume fractions of the primary NiAl-precipitate in FBB8 (16 ~ 18%) are determined largely by the amount of Ni and Al^24^. In the current study, the addition of Ti (2 and 4 wt%) into FBB8 gives rise to volume fractions of the primary precipitates (16 ~ 22%), comparable to those of FBB8. As Ti partitions preferentially into the primary precipitates (Table 1), the two-phase decomposition (B2 and L2_1_) within the primary precipitate occurs for the 2-wt%-Ti alloy, whereas complete ordering from the B2 to L2_1_ structures occurs in the precipitates of the 4-wt%-Ti alloy. It has been reported that similar hierarchical structures can be formed by controlling the Ti content in Fe-Cr-Ni-Al-Ti alloys^42^,^43^. Liebscher *et al.* suggested the heterogeneous nucleation of B2 zones on L2_1_ anti-phase domain boundaries as a possible mechanism for the formation of these hierarchical structures in precipitates^43^. A systematic study of the effect of the Ti content on the precipitate structure in the current alloys is ongoing and will be published elsewhere.

As a comparison, our earlier work investigated the evolution of interphase and intergranular lattice strains of FBB8 during *in-situ* tension and creep experiments up to 973 K, using ND^33^,^53^. In this case, no clear load transfer from the matrix to the B2 precipitate was observed, owing to the diffusional flow along the matrix-precipitate interface^54^, which leads to the ineffective load transfer^53^. Thus, the B2 precipitate alone was not an effective reinforcing media at elevated temperatures. The ND results in the present study demonstrate that the hierarchical NiAl/Ni_2_TiAl precipitates can effectively assume the load from the Fe matrix during loading and creep deformation (Fig. 3), which indicates that the diffusional flow along the matrix-precipitate interface is less significant in HPSFA, relative to the FBB8, resulting in the superior creep resistance of HPSFA (Fig. 2a,b). Besides dislocation climb and glide, high-temperature deformation can occur via the diffusional flow accommodated by grain-boundary sliding, this latter mechanism depending on grain size^55^,^56^,^57^,^58^,^59^. Because the grain size of the present HPSFA is large (estimated > 200 μm), the grain-boundary sliding and diffusional flow are not expected to be dominant, as compared to the dislocation creep, especially given the relatively-low homologous temperature and high stresses. However, we cannot exclude that slight decreases of the Fe matrix lattice strain with increasing the creep time at 190 MPa (Fig. 3b) may be due to a combination of dislocation and diffusional creep. Future experiments where the grain size is changed systematically (e.g., by grain growth during homogenization) will be able to shed light on this issue.

**Figure 4:**
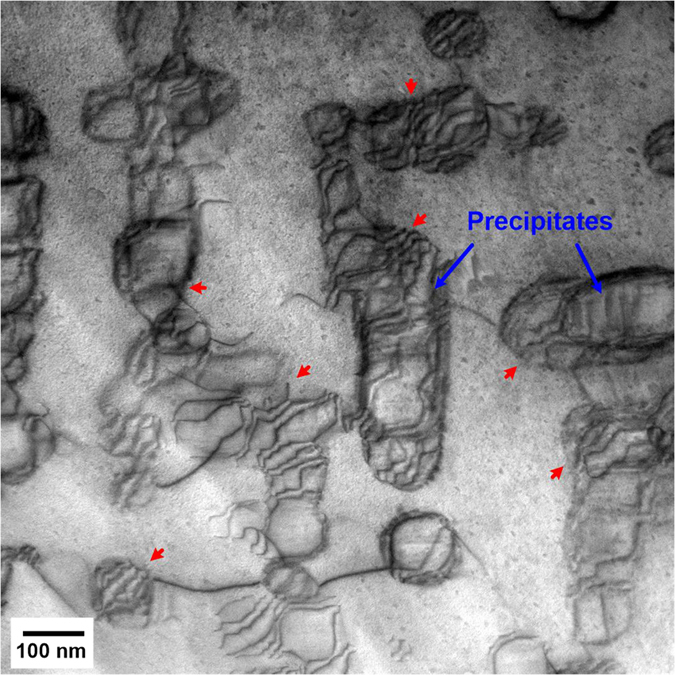
STEM image of the crept HPSFA. A bright-field (BF) scanning-transmission-electron-microscopy (STEM) image of an HPSFA sample crept at 140 MPa and 973 K, which was interrupted by cooling down to room temperature under the applied stress at the creep time of 200 hours (red arrows: dislocations, blue arrows: precipitates).

The coarsening behavior of the precipitates is significant for the long-term creep properties^60^. It has been shown that the low mismatch between the precipitate and the matrix in FBB8 is effective in suppressing the structural change, and, thus, leads to the excellent resistance to precipitate coarsening during exposure to elevated temperatures^24^,^37^. The temporal evolution of the precipitate size of HPSFA at 973 K is observed to be comparable to that of FBB8 (Supplementary Figure S4), demonstrating the comparable coarsening resistance of HPSFA at 973 K. Moreover, a large number of interfacial dislocations form at the matrix/precipitate interface on the crept sample of HPSFA at 140 MPa and 973 K for 200 hours, as indicated by blue arrows in Fig. 4. Since the specimen under the standard heat treatment is devoid of misfit dislocations, the interfacial dislocation in Fig. 4 is formed during creep deformation, which was not observed in NiAl-strengthened alloys^37^. The misfit strain at the interface between the matrix and NiAl/Ni_2_TiAl precipitates of HPSFA could lead to an attractive force due to the strain fields of precipitates and dislocations^45^. Thus, more dislocations will be trapped at the interfaces, and the density of the mobile dislocations decreases^45^. However, SPSFA contains a high density of interfacial dislocations in the aged condition, as shown in Fig 1c, which reduces the misfit strain at the interface. This reduction in the coherency misfit strain and the presence of interfacial dislocations allowing pipe diffusion in SPSFA may reduce the effectiveness of the precipitates as obstacles against matrix dislocations by shearing and/or climb-bypass^45^, thus increasing the creep rate of the alloy, relative to HPSFA (Fig. 2a,b).

**Figure 5:**
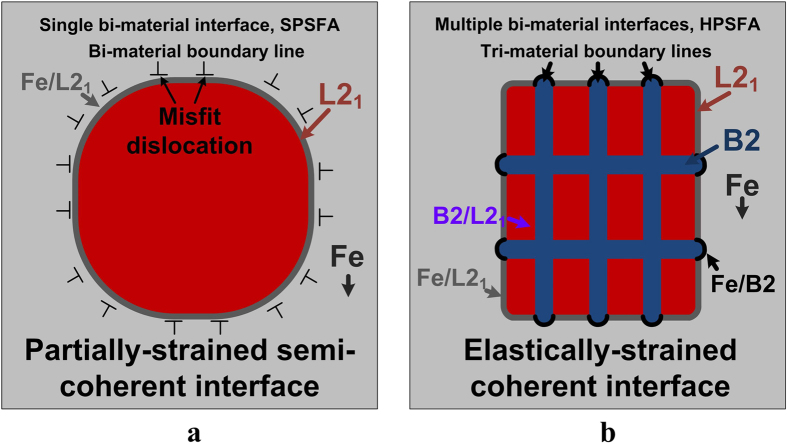
Schematic illustration of the as-aged microstructure of SPSFA and HPSFA. Schematics illustrating the distinct strain fields of the as-aged microstructure before creep deformation, depending on the interface structures of the precipitates. (**a**) A single bi-material precipitate-matrix interface of SPSFA. (**b**) Multiple tri-material interfaces within the precipitate and between the precipitate and matrix of HPSFA.

The lattice mismatch between the Fe and B2/L2_1_ phases of HPSFA cannot be accurately determined from the present ND measurements, due to the low volume fraction and structural similarity of the B2 and L2_1_ phases (Supplementary Figure S2). The lattice mismatch is defined as


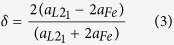


where *δ* is the lattice mismatch, and 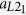 and 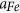 are the lattice constants of the L2_1_ and Fe phases, respectively. Since the L2_1_ structure consists of eight sub-lattices of a B2 structure, the lattice parameter of the Fe was multiplied by a factor of two for the corresponding structural comparison. Assuming that the Fe and L2_1_ phases only contribute to the ND intensity, the lattice constants of the Fe and L2_1_ phases were derived and are 2.8894 and 5.8224 Å at room temperature, respectively, which correspond to a misfit of 0.7%. In contrast, the reasonable intensity of the L2_1_ phase superlattice and well-separated fundamental reflections of SPSFA made more accurate ND analyses possible (Supplementary Figure S2c). The lattice constants of the Fe and L2_1_ phases of SPSFA were determined, using the Rietveld full pattern refinements^48^. They are 2.8864 and 5.8537 Å at room temperature, respectively, which correspond to a lattice misfit of 1.3%. The compositions of the Fe and L2_1_ phases of HPSFA, determined by atom-probe tomography (APT), are almost identical to those of the Fe and L2_1_ phases of SPSFA, derived from the TEM EDS analysis (Table 1), and, thus, the lattice mismatch between the Fe and L2_1_ phases of HPSFA is believed to range from 0.7 to 1.3%. Our previous study on the microstructure of FBB8 using APT and ND reveals that the lattice mismatch between the B2 precipitate and Fe matrix is near zero^24^. Similarly, the composition of the B2 phase of HPSFA, obtained from APT (Table 1), is close to that of FBB8^24^. Thus, it is also believed that the lattice mismatch between the B2 precipitate and Fe matrix of HPSFA is near zero. The TEM observation of the interface morphology among the Fe, B2, and L2_1_ phases in Fig. 1b also supports this approximation: flat interfaces of Fe/L2_1_ and L2_1_/B2 (high misfit strain), and round-like interfaces of Fe/B2 (low misfit strain), which is also reflected in the schematic of Fig. 5.

The morphology of the coherent precipitates strongly depend on the misfit strain, interfacial energy, and size of the precipitate^32^. With a high level of the misfit strain caused by the lattice mismatch between the matrix and precipitate, the stable shape of the coherent precipitates becomes cuboidal, as observed in HPSFA (Figs 1b and 5b). However, as the size of the precipitate and the lattice mismatch increase, the misfit strain increases accordingly. When the misfit strain becomes higher than the critical level that the microstructure can accommodate, misfit dislocations will form to release the excessive misfit strain at the interface (semi-coherent)^61^, which leads to a transition to an elliptical shape with the reduced misfit strain. This trend is observed in SPSFA with a higher lattice mismatch (1.3%) and larger precipitate size (220 nm) (Figs 1c and 5a) than HPSFA (lattice mismatch: 0.7%, precipitate size: 90 nm), (Figs 1b and 5b). In contrast, the HPSFA with 2 wt% Ti exhibits hierarchical precipitates with a lamellar structure of B2/L2_1_ phases. It has been reported that such a lamellar structure in the coherent precipitate can lower the elastic strain at the interface^62^. Moreover, the hierarchical precipitate structure with tri-material interfaces in the 2-wt%-Ti alloy, consisting of individual bi-material interfaces separated by tri-material boundary lines (Fig. 5b), may play an important role in maintaining the misfit strain at a more complex series of interfaces, thus retaining the full coherency of the precipitates. Therefore, we hypothesize that the hierarchical precipitates with the tri-material interfaces in the 2-wt%-Ti alloy is particularly effective at retaining the coherency of the misfitting precipitates.

The creep resistance of alloys containing coherent, misfitting precipitates is known to increase as the magnitude of the precipitate/matrix lattice mismatch increases, thus increasing the elastic-strain field created in the matrix by the precipitates^63^: This effect also becomes more pronounced, as the precipitate size increases^64^,^65^ and is explained by the fact that the elastic field in the matrix created by mismatching precipitates enhances the elastic interaction between the precipitate and mobile dislocations^66^, reducing the climb rate of the dislocations bypassing the precipitates, and, hence, decreasing the creep rate of the alloy. Similarly, the 2-wt%-Ti alloy with a hierarchical structure phase contains coherent precipitates with a cuboidal morphology. This morphology indicates a high level of elastic mismatch strains^67^,^68^. Therefore, a high level of elastic interactions can be expected between the precipitates and dislocations; we believe that this trend explains the high creep resistance of this alloy, but modeling needs to confirm the magnitude of this mechanism, thus, opening the door to a prediction of the optimal concentration of Ti in these alloys.

**Figure 6:**
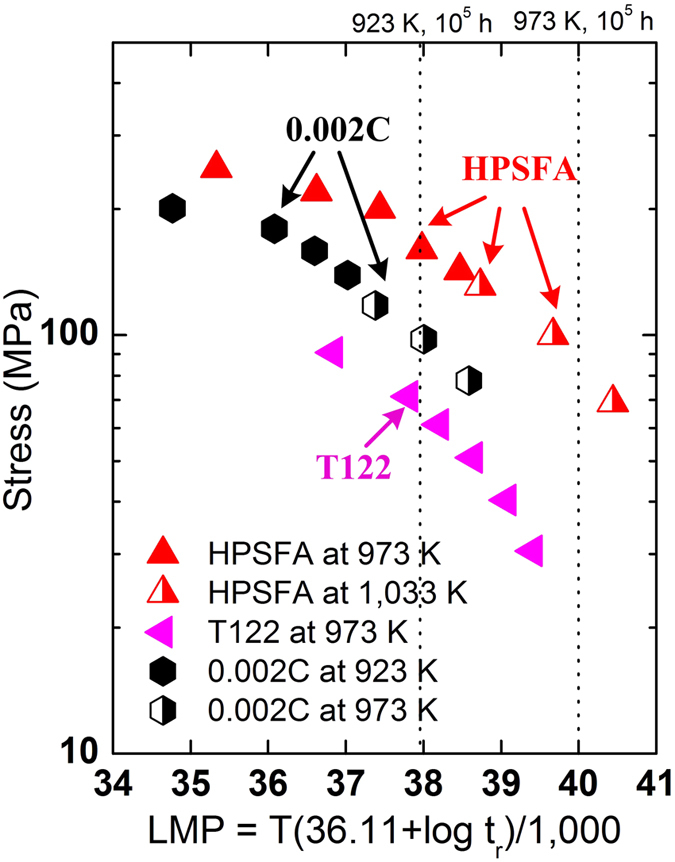
The determination of creep strengths with Larson-Miller parameter. Larson-Miller plot for HPSFA, T122, and 0.002C steels^3^,^36^, the LMP values at 923 and 973 K for 100,000 hours are indicated by dotted lines in the plot.

The creep strength at 923 and 973 K for 10^5^ hours is estimated, using the Larson-Miller parameter (LMP)^69^, which is compared to the creep strength for the available conventional ferritic steels (T122 and 0.002C)^3^,^36^. The LM parameter is defined as P_LM_ = T (log t_r_ + C), where T is the absolute temperature in Kelvin, t_r_ is the time to creep rupture in hour, and C is a LM constant. Figure 6 presents the LM parameter of HPSFA as a function of stress with C = 36.11, which was employed for high-Cr ferritic steels, such as T122 and 0.002C^3^,^36^. Since T122 and 0.002 C steels possess the best creep resistance among the available conventional ferritic steels^3^,^36^, the LM parameters of the T122 and 0.002C steels are also included for comparison in Fig. 6. The creep strength was calculated to be 164 MPa at 923 K for HPSFA, about 64 MPa higher than that of the 0.002C steel (100 MPa), which is the most creep resistant among the available conventional ferritic steels^3^. The calculated creep strength at 973 K was 89 MPa for HPSFA, about five times higher than that (18 MPa) for T122^36^, which is one of the most creep-resistant steels compared in Fig. 2b. Moreover, the yield strength at 973 K of HPSFA is two times higher (280 MPa) than FBB8 (120 MPa), which also indicates the superior high-temperature properties of HPSFA (Table 2). The considerable increase of the creep and tension strengths implies the possibility of the HPSFA alloy to be used for higher temperatures, such as 1,033 K, which is the currently-required temperature for the USC steam turbines^20^.

Overall, a novel hierarchical ferritic alloy was developed by introducing coherent L2_1_-type precipitates strengthened by nano-scaled B2 zones into the Fe matrix. Systematic investigations were conducted, using an integrated experimental (TEM/STEM, *in-situ* ND, and APT) and theoretical (crystal-plasticity finite-element modeling) approach. The experimental results reveal that the hierarchical structure gives rise to coherent interfaces between the Fe and precipitate phases with optimized misfit strains, which leads to the excellent creep resistance at 973 K. From the micromechanical modeling of the lattice strain, the load-partitioning mechanism is found to be load transferring from the Fe matrix to the hard precipitate, which indicates the insufficient diffusional flow along the matrix/precipitate interface and a strong interaction between the matrix and mobile dislocations. This trend is in sharp contrast to the behavior of FBB8 at 973 K. These results could provide a new alloy-design strategy, accelerate the advance in the development of creep-resistant alloys, and broaden the applications of ferritic alloys to higher temperatures.

## Methods

### Materials

The nominal composition of the alloys is Fe-6.5Al-10Cr-10Ni-xTi-3.4Mo-0.25Zr-0.005B with x = 2 and 4 in wt%. An ingot of the 2%-Ti alloy with a dimension of 12.7 × 25.4 × 1.9 cm^3^ and a rod ingot of the 4%-Ti alloy with 2 kg and a diameter of 5.08 cm were prepared by Sophisticated Alloys, Inc., using the vacuum-induction-melting facility. Hot isostatic pressing (HIP) was applied to the ingots at 1,473 K and 100 MPa for 4 hours in order to reduce defects forming during the casting and cooling processes. These alloys were homogenized at 1,473 K for 30 minutes, followed by air cooling and, then, aged at 973 K for 100 hours.

### Tension and Creep Experiments at Elevated Temperatures

Two types of tension-creep samples were machined, (a) a round type with a gage diameter of 3.175 mm and a gage length of 28 mm and (b) a dog-bone type with a cross section of 3 mm × 3 mm and a gage length of 25 mm. Tension-creep tests were conducted at 973 and 1,033 K under a constant load. Samples for tension-creep tests on HPSFA at 1,033 K (Fig. 6) were subjected to the solution treatment at 1,473 K for 30 minutes, followed by the aging treatment at 1,073 K for 5 hours, which gives a similar size and morphology of the precipitate of the HPSFA aged at 973 K for 100 hours. Step-loading compressive creep tests were conducted at 973 K in which an 8-mm-diameter ×16-mm-height sample was loaded at a constant load until a steady-state creep rate was reached, and, then, the applied load was increased, and so on. Tension tests with a round type sample (a gage diameter of 3.175 mm and a gage length of 28 mm) at 973 K were conducted at the strain rate of 10^−4^ s^−1^, using the hydraulic Materials Testing System (MTS) machines (Table 2). A thermocouple was attached to the center of the specimen gauge-length section. The sample was heated to and held at 973 K for at least 0.5 hour until the sample temperature is stabilized at 973 K within ±10 K.

### Microstructural Characterization

Scanning-electron microscopy (SEM) was conducted, using a Zeiss Auriga 40 equipped with an Everhart-Thornley secondary-electron detector. The SEM images were analyzed, using the ImageJ software^70^ to obtain the sizes and volume fractions of the precipitates, and the averaged values were estimated, using more than 200 particles. The thin foils for scanning-transmission-electron microscopy (STEM) and conventional transmission-electron-microscopy (CTEM) observations were prepared by electropolishing, followed by ion milling at the ion energy of ~2 kV and an incident angle of ± 6 degree. The TEM specimens were cooled by liquid N_2_ during ion milling. The STEM observations were performed with a JEOL JEM-2100F TEM equipped with double spherical aberration correctors for probe-forming and image-forming lenses. The high-angle annular dark-field (HAADF) STEM images were acquired, using a detector-collection angle ranging from 100 to 267 mrad, while the bright-field (BF) STEM images were simultaneously recorded, using a STEM BF detector.

The sharp-tip specimens for atom-probe tomography (APT) were prepared in a FEI Nova 200 equipped with a dual-electron-beam and focused-ion-beam (FIB) column. The APT data acquisition was conducted, using a CAMECA local electrode atom probe (LEAP), 4000X HR, equipped with an energy-compensated reflectron lens. The APT measurements were performed in both voltage and laser modes to validate the precipitate compositions and sizes in both modes. The data-acquisition temperature was set to 50 K, and the pulse frequency and fraction were 200 kHz and 20%, respectively, for the voltage-mode runs. The temperature was set to 30 K, and the laser energy was set to 100 pJ for the laser-pulsed runs. The TEM energy-dispersive X ray spectroscopy (EDS) was conducted, using a Zeiss Libra 200 MC TEM/STEM equipped with an EDS detector, a Bruker X-Flash 5030. The X-ray collection time was between 300 and 500 s, and at least 10 single measurements were obtained. The EDS compositions were averaged over the 10 measurements.

### Neutron Diffraction

The *in-situ* neutron-diffraction (ND) experiments were carried out on the Spectrometer for MAterials Research at Temperature and Stress (SMARTS) diffractometer of the Los Alamos Neutron Science Center (LANSCE) facility located at the Los Alamos National Laboratory^71^. The ND instrument utilizes time-of-flight (TOF) measurements, in which the incident beam is polychromatic with a range of wave lengths, which allows for the ND measurements with a diffraction pattern covering a wide range of d spacings without the rotation of samples or detectors. Two detectors, which are fixed at an angle of 45° to the loading direction, were employed to collect the diffracted beams from polycrystalline grains with lattice planes parallel to the transverse and axial directions, respectively. Therefore, the lattice parameters of differently-oriented grains and each of the phases can be measured simultaneously both parallel and perpendicular to the loading directions^33^,^72^. Screw-threaded cylindrical samples with a gage diameter of 6.35 mm and a gage length of 40 mm were machined for the *in-situ* creep experiments. *In-situ* creep tests were performed in vacuum at 973 K under constant load levels. Before the *in-situ* creep test, the lattice parameter was measured at elevated temperatures. The samples were studied by ND at high temperatures after the sample saturated within ± 1 K of the target temperature, and the sample displacement was equilibrated. The ND data were collected for 15 minutes. After the ND measurement at 973 K, *in-situ* creep experiments were performed under constant load levels, and the macroscopic strain was measured, using a high-temperature extensometer over a gauge length of 25 mm within a measureable strain range of 10%. The sample was subjected to constant stress levels of 100, 150, 190, 220, and 235 MPa. After the stress was increased to 235 MPa, the sample fractured in one hour. The ND data were collected in 10-minute increments. The total creep strain to final rupture was about 9%, which is within the measureable strain range of the extensometer used.

### Finite-Element Crystal-Plasticity Model

The microstructure-based finite-element simulations use the crystal-plasticity model, which is implemented as a user material subroutine in the commercial software ABAQUS^50^. The constitutive relationship used in the simulations was defined through the Peirce-Asaro-Needleman power law given by,


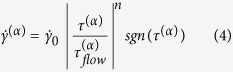


where 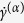 is the slip strain rate, 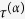 is the resolved shear stress, 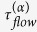 represents the flow strength of the 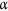-th slip system, and 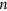 is the stress exponent. Details of the hardening law and other constitutive parameters can be found in the Supplementary Table S2^73^. A 15 × 15 × 15 element cubic model was set up (Fig. 3c), and divided into 125 grains with each of 27 elements making up a 3 × 3 × 3 cubic grain. The 5 elements were randomly selected and assumed to be the L2_1_ precipitates in each grain. The volume fraction of the L2_1_ precipitates, compared to the Fe matrix, was set to be 18.5%. A second type of B2 precipitates was introduced with an element smaller in size embedded in the pre-existing L2_1_ elements in each grain. The B2 elements were set to have a volume fraction of 50%, compared to L2_1_ elements, and 9.26%, relative to the Fe matrix. In essence, each 27-element grain contained a total of 22 elements with the Fe matrix crystal-plasticity parameters assigned to them. The remaining 5 elements each contain 6 trapezoidal elements at the 6 faces of the cubic element with a smaller cubic elements attached at the center of these 5 elements. The trapezoidal elements were assumed to have the L2_1_-precipitate crystal-plasticity parameters, and the smaller cubic elements were assumed to have B2-precipitate properties.

## Additional Information

**How to cite this article**: Song, G. *et al.* Ferritic Alloys with Extreme Creep Resistance via Coherent Hierarchical Precipitates. *Sci. Rep.* **5**, 16327; doi: 10.1038/srep16327 (2015).

## Supplementary Material

Supplementary Information

## Figures and Tables

**Table 1:** APT and TEM-EDS compositions of HPSFA and SPSFA.

Alloy	Tool	Phase	Al	Fe	Ni	Ti	Mo	Cr
HPSFA	APT	L2_1_	29.253 ± 0.230	18.057 ± 0.188	36.235 ± 0.209	15.399 ± 0.188	0.466 ± 0.037	0.591 ± 0.048
		B2	38.206 ± 0.039	14.030 ± 0.028	42.826 ± 0.037	4.428 ± 0.015	0.075 ± 0.003	0.432 ± 0.005
		Fe	7.096 ± 0.018	75.859 ± 0.033	1.548 ± 0.009	0.407 ± 0.005	2.283 ± 0.010	12.807 ± 0.026
SPSFA	TEM EDS	L2_1_	25.0 ± 2.0	22.9 ± 3.7	33.8 ± 1.9	16.5 ± 0.9	0.1 ± 0.1	1.0 ± 0.7
		Fe	7.0 ± 0.4	74.9 ± 0.5	2.1 ± 0.2	1.0 ± 0.1	2.0 ± 0.2	12.8 ± 0.2

Chemical compositions (in at%) of constitutive phases in HPSFA and SPSFA, determined using the atom-probe tomography (APT) and transmission-electron-microscopy (TEM) energy-dispersive X-ray spectroscopy (EDS), respectively. Both HPSFA and SPSFA specimens were aged at 973 K for 100 hours. The uncertainties for the EDS results are represented by the standard deviation from the measurement series, while those of APT represent the statistical counting scatter^74^.

**Table 2:**
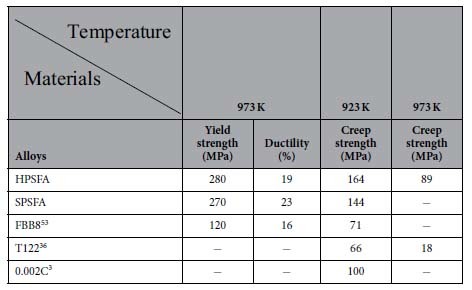
Comparison of mechanical properties at high temperatures.

A summary of mechanical properties at elevated temperatures of HPSFA, SPSFA, and FBB8, such as tension-yield strength/ductility at 973 K, and creep strength for 100,000 hours at 923 and 973 K. The creep strengths of 0.002C and T122 steels are also included for comparison^3^,^36^. All the tension and creep samples of HPSFA, SPSFA, and FBB8 at 973 K were aged at 973 K for 100 hours, while HPSFA specimens for creep tests at 1,033 K were aged at 1,073 K for 5 hours (See the Methods Section: Materials).
