# Supplementary material for: Ferritic Alloys with Extreme Creep Resistance via Coherent Hierarchical Precipitates
Source: Sci Rep. 2015 Nov 9;5:16327. doi: 10.1038/srep16327 (PMC4637877; doi:10.1038/srep16327)
Supplement: Supplementary Information [file srep16327-s1.pdf]

Supplementary Information

Ferritic Alloys with Extreme Creep Resistance via Coherent Hierarchical Precipitates.

Gian Song[1], Zhiqian Sun[1], Lin Li[1], Xiandong Xu[2], Michael Rawlings[3], Christian H. Liebscher[4], Bjørn Clausen[5], Jonathan Poplawsky[6], Donovan N. Leonard[6], Shenyan Huang[1], Zhenke Teng[1], Chain T. Liu[7], Mark D. Asta[4], Yanfei Gao[1], David C. Dunand[3], Gautam Ghosh[3], Mingwei Chen[2], Morris E. Fine[3], and Peter K. Liaw[1*].

1. Department of Materials Science and Engineering, The University of Tennessee, Knoxville, TN, 37996-2200
2. WPI Advanced Institute for Materials Research, Tohoku University, Sendai 980-8577, Japan
3. Department of Materials Science and Engineering, Northwestern University, Evanston, IL 60208-3108
4. Department of Materials Science and Engineering, University of California, Berkeley, CA 94720
5. Lujan Center, Los Alamos National Laboratory, Los Alamos, NM 87545, USA
6. Center for Nano phase Materials Sciences, Oak Ridge National Laboratory, Oak Ridge, TN 37831, USA
7. Center for Advanced Structural Materials, Department of Mechanical and Biomedical Engineering, City University of Hong Kong, Kowloon, Hong Kong.

*Correspondence to pliaw@utk.edu

- ***Transmission-Electron Microscopy***

Figure S1a exhibits the selected-area-diffraction pattern (SADP) of the [101] zone axis with superlattice reflections of the $L2_1$ structure. Figures S1b and c are the dark-field (DF)-TEM images acquired on the same region, using different superlattice reflections. Figure S1b is a DF-TEM image acquired, using the <111> reflection, and exhibits the narrow dark zones within the $L2_1$ precipitate. The DF-TEM image in Figure S1c, using the <020> reflection, reveals narrow zones within the parent $L2_1$ phase, showing a higher intensity than the surrounding $L2_1$-precipitate phase. The DF-TEM image, using the <111> reflection in Figure S1b, which is unique to the $L2_1$ structure, exhibits the possible presence of anti-phase boundaries (APBs) or a second phase (B2-NiAl) within the $L2_1$ phases. Moreover, the DF-TEM image, using the <020> reflection in Figure S1c, which is common to both phases, presents the same narrow zones, but brighter contrast than those originating from the $L2_1$ precipitates. Since APBs should be invisible, when imaged using the <020> or <222> reflections[1], and the B2-NiAl has a higher structure factor, relative to the $L2_1$ structure[2], the bright contrast zones in Figure S1c are considered as the B2 phases.

- ***ND Experiment***

Figure S2 shows the representative ND patterns of (a) and (c) SPSFA and (b) and (d) HPSFA measured at room temperature without loading (a reference state, 5 MPa), refined by the General Structural Analysis System (GSAS) Rietveld analysis[3]. The ND patterns of SPSFA and HPSFA contain fundamental matrix/precipitate reflections (e.g., 110, 200, 211, and 220 peaks), and low intensity superlattice peaks (e.g., 111, 200, 222, 311, and 420 peaks) from the $L2_1$ precipitate, as shown in Figures S2a and b. The enlarged pattern of SPSFA in Figure S2c clearly reveals that the fundamental peaks of Fe and $L2_1$ phases are well-separated. In contrast, the ND pattern of HPSFA exhibits that the fundamental peaks of Fe

and $L2_1$ phases are overlapped, as shown in the Figure S2d. Since HPSFA consists of a $L2_1$ phase as a parent precipitate surrounding a small fraction of a B2 phase, as shown in the TEM results (Figures 1b, 1c, and Figure S1), the superlattice reflections are believed to originate from the parent $L2_1$ phase, although ND cannot detect the superlattice reflections (e.g., 111 and 311 peaks, which are unique to the $L2_1$ structure) due to the limited intensity of neutrons. Moreover, the ND with the limited resolution appeared to be incapable of detecting the B2 phase. Thus, the Rietveld refinement[3] was conducted with an assumption of the presence of the Fe matrix and $L2_1$ phases for both HPSFA and SPSFA.

The averaged phase strain represents the volume-averaged lattice strain of the individual phase (Fe or $L2_1$), which depends on the elastic and plastic anisotropy of the individual phases. In order to obtain the averaged phase strain, a whole-pattern Rietveld refinement was performed to fit the entire ND spectrum, employing the GSAS Program developed at the Los Alamos National Laboratory[3]. The average phase strain is calculated, using the following formula

$$\varepsilon = (a - a_0)/a_0 \qquad \text{(S1)}$$

where a is the lattice parameter of a given phase measured during heating and/or loading, and a_0 is the corresponding lattice parameter before loading (5 MPa at 973 K). The lattice parameters extracted from the Rietveld-refinement approach were utilized for the misfit calculations, which is defined as

$$\delta = \frac{2(a_{L2_1} - 2a_{Fe})}{(a_{L2_1} + 2a_{Fe})} \qquad \text{(S2)}$$

where δ is the lattice misfit, and a_{Fe} and a_{L2_1} are the lattice parameters of Fe and $L2_1$ phases, respectively.

Lattice parameters of the precipitate and matrix were determined at room temperature, using the Rietveld refinement[3]. Note that the lattice parameter of the $L2_1$ structure phase is about 2 times larger than that of the BCC Fe structure, since a $L2_1$ structure consists of eight sub-lattices of a BCC structure[4]. The lattice parameters of the Fe and $L2_1$ phases at room temperature for SPSFA are 2.8864 and 5.8537 Å, while those of HPSFA are 2.8894 Å and 5.8224 Å, respectively. The lattice misfit between the matrix and precipitate phases at room temperature was calculated, using Eq. (S2), to be 1.3 % for SPSFA and 0.7 % for HPSFA, respectively.

- ***Finite-Element Crystal-Plasticity Model***

The crystal-plasticity constitutive relationship and the lattice-strain evolution defined in the present simulation are discussed below. The kinematics is described, using the multiplicative decomposition:

$$F_{ij} = F^{e}_{ik}F^{p}_{kj} \quad \text{where} \quad F_{ij} = \frac{dx_i}{dX_j} \tag{S3}$$

In this case, F^{e}_{ik} represents the elastic portion, and F^{p}_{kj} stands for the plastic portion of the total deformation gradient, F_{ij}. The elastic-constitutive behavior is described, using the following relationship,

$$T_{ij} = C_{ijkl}E^{e}_{kl} \tag{S4}$$

In this case, E^{e}_{kl} is the Lagrange-Green strain, and T_{ij} represents the material-stress tensor, which is related to the Cauchy stress, σ_{ij}, through the following relationship,

$$J\sigma_{ij} = F^{e}_{ik}T_{kl}F^{e}_{jl} \quad \text{where} \quad J = \det(F^{e}) \tag{S5}$$

As for the plastic portion, F^{p}_{kj}, the following relationship was considered,

$$\dot{F}^{p}_{ik}F^{p-1}_{kj} = \sum_{\alpha=1}^{NSLIP} \dot{\gamma}^{(\alpha)} s_i^{(\alpha)} m_j^{(\alpha)} \tag{S6}$$

where NSLIP is the total number of slip systems, $\dot{\gamma}^{(\alpha)}$ is the strain rate of slip, and $s_i^{(\alpha)}$ and $m_j^{(\alpha)}$ represent the slip direction and slip plane normal, respectively, of the α-th system.

The hardening law employed in this model is expressed in terms of,

$$\dot{\gamma}^{(\alpha)} = \dot{\gamma}_0 \left|\frac{\tau^{(\alpha)}}{\tau^{(\alpha)}_{flow}}\right|^{n} sgn(\tau^{(\alpha)}) \tag{S7}$$

$$\dot{\tau}^{(\alpha)}_{flow} = \sum_\beta h_{\alpha\beta}|\dot{\gamma}^{(\beta)}| \text{ where } h_{\alpha\beta} = h(\gamma)[q + (1-q)\delta_{\alpha\beta}], \text{ when } \alpha \neq \beta$$

However, for the self-hardening model, we use,

$$h_{\alpha\alpha} = h(\gamma)\, h_0\, sech^2 \left|\frac{h_0\gamma}{\tau_s - \tau_0}\right| \tag{S8}$$

where h_0 is the initial hardening modulus, τ_0 is the initial slip strength, and τ_s is the saturated slip strength. $\dot{\gamma}_0^{(\alpha)}$ is the characteristic strain rate, n is the stress component, and $h_{\alpha\beta}$ is the latent hardening moduli. The terms, $\tau^{(\alpha)}$ and $\tau^{(\alpha)}_{flow}$, are the resolved shear stress and flow strength of the α-th slip system, respectively, n is the stress exponent, and q is the latent-hardening coefficient in the same set of slip systems.

The orientations of each Fe grain were set to be random within the setup of the model. In order to simulate the lattice-strain behavior and compare that to the experimental results obtained from the ND results, a certain set of grains, which have the orientation, <hkl>, whose degree angles are within a certain range of error margins from the diffraction vector, Q, was selected. This set of grains is normally about 2 % of the total grains, and the orientation error margin is normally set to be ± 5°, compared to the vector, Q[5].

The thermal-residual simulation includes a thermally-heating model from room temperature up to 1,023 K, followed by applying a uniaxial stress of 650 MPa. The pre-existing thermal stress will generate a residual stress within the model, therefore resulting in a differentiated starting point for the lattice strain in the stress versus lattice-strain diagram (e.g., Figure 3e). The beginning points were, then, adjusted to be starting at the origin again for the purpose of the comparison with the pure loading scenario. Note that all the phases (Fe, B2, and $L2_1$ phases) at zero strain are subjected to the thermal stress caused by the difference in the thermal expansion between three phases (about 16 MPa) in Figure 3e.

References

1. Hirsch, P. B., Howie, A., Nicholson, R. B., Pashley, D. W. & Whelan, M. J. *Electron microscopy of thin crystals* (Plenum Press, New York, 1965).
2. Liebscher, C. H., Radmilovic, V., Dahmen, U., Asta, M. & Ghosh, G. On the formation of hierarchically structured $L2_1$-Ni_2TiAl type precipitates in a ferritic alloy. *Journal of Materials Science* **48**, 2067-2075, (2013).
3. Larson A. C., Von Dreele R. B. *General structure analysis system (GSAS). Los Alamos National Laboratory Report LAUR*, 86-748, (1994).
4. Strutt, P., Polvani, R. & Ingram, J. Creep behavior of the heusler type structure alloy Ni_2AlTi. *Metallurgical and Materials Transactions A* **7**, 23-31, (1976).
5. Huang, S., Computational and experimental study of structure-property relationships in NiAl precipitate-strengthened ferritic superalloys. *Ph.D. thesis: The University of Tennessee, Knoxville*, (2011)
6. Sun, Z., Song G., Ilavsky J., Ghosh G., and Liaw, P. K., The nano-sized precipitate stability and its controlling factors in a NiAl-strengthened ferritic alloy, Submitted.
7. Taneike, M., Abe, F. & Sawada, K. Creep-strengthening of steel at high temperatures using nano-sized carbonitride dispersions. *Nature* **424**, 294-296, (2003).
8. Kimura, K., Sawada, K., Kushima, H. & Toda, Y. *in Challenges of power engineering and environment* (eds, Kefa Cen, Yong Chi, & Fei Wang) Ch. 196, 1059-1065 (Springer Berlin Heidelberg, 2007).
9. Yoshizawa, M., Igarashi, M., Moriguchi, K., Iseda, A., Armaki, H. G. & Maruyama, K. Effect of precipitates on long-term creep deformation properties of P92 and P122 type advanced ferritic steels for USC power plants. *Materials Science and Engineering: A* **510–511**, 162-168, (2009).

10. Sawada, K., Kubo, K. & Abe, F. Creep behavior and stability of MX precipitates at high temperature in 9Cr–0.5Mo–1.8W–VNb steel. *Materials Science and Engineering: A* **319–321**, 784-787, (2001).

11. Masuyama, F. & Komai, N. Evaluation of long-term creep rupture strength of tungsten-strengthened advanced 9-12% Cr steels. *Key Engineering Materials* **171**, 179-188, (2000).

Figure Legends

Figure S1 **DF-TEM characterization of HPSFA**. A selected-area-diffraction-pattern (a) and false color dark-field (DF)-TEM images acquired along the [101] zone axis from the same region, using (b) <111> and (c) <020> super-lattice reflections, respectively, for the HPSFA alloy subjected to the solution treatment at 1,473 K for 30 minutes, followed by aging at 973 K for 100 hours.

Figure S2 **Comparison of the ND results between SPSFA and HPSFA.** Representative neutron-diffraction patterns (intensity vs. D spacing; plane distance) of (a) SPSFA and (b) HPSFA measured at room temperature without loading. Enlarged patterns in (c) and (d) clearly exhibit well-separated and overlapped fundamental $(110)_{Fe}$ and $(220)_{L21}$ peaks for SPSFA and HPSFA, respectively. The red cross represents the measured data. The green curve is the fitted profile using the GSAS Rietveld analysis[3]. The pink curve presents the difference between the fitted profile and measured data. The red and black toggles below the patterns represent the peak positions of the $L2_1$ and BCC-Fe phases, which are determined by the phase information in the GSAS program, such as the space group and lattice parameter, respectively.

Figure S3 **Comparison of the creep behavior between FBB8 and HPSFA.** Creep-strain-rate versus time at 973 K, 100 MPa for FBB8 and 160 MPa for HPSFA, respectively. It can be observed that the creep-deformation process of HPSFA is composed of the well-defined secondary-creep region, while an extended tertiary creep accounts for much of the creep life in FBB8 after the primary creep where the creep rate gradually decreases.

Figure S4 Temporal evolution of the precipitate sizes of HPSFA and FBB8 at 973 K. Note that the HPSFA specimens were aged at 973 K for 100 hours, followed by creep tests, and the precipitate sizes of HPSFA were derived from the grip sections of the crept samples at 973 K (no stress). The FBB8 specimens were aged at 973 K as a function of time[6]. Since the precipitate of HPSFA is of an elongated shape, as observed in Figures 1b, 1c, 5b, and Figures S1b, and S1c, the width and length of the precipitate were separately determined. In contrast, since the precipitate of FBB8 has a spherical morphology, as observed in Figure 1a, the diameter of the precipitate was employed.

Table S1 Bulk chemical compositions of conventional ferritic steels. Chemical compositions (in wt. %) of the conventional ferritic steels compared for the creep resistance in Figures 2 and 6[7-11].

Table S2 Summary of parameters employed in the elastic-plastic constitutive law. C_{11}, C_{12}, and C_{44}: Elastic constants, n: stress component, q: latent-hardening coefficient in the same set of slip systems, h_0: initial hardening modulus, τ_s: saturated slip strength, τ_0: initial slip strength, and α: slip-system number[5], See the Supplementary Information Section: Finite-Element Crystal-Plasticity Model.

Correspondences and requests for materials should be addressed to the corresponding and first authors.

(e-mail: pliaw@utk.edu and gsong1@vols.utk.edu)

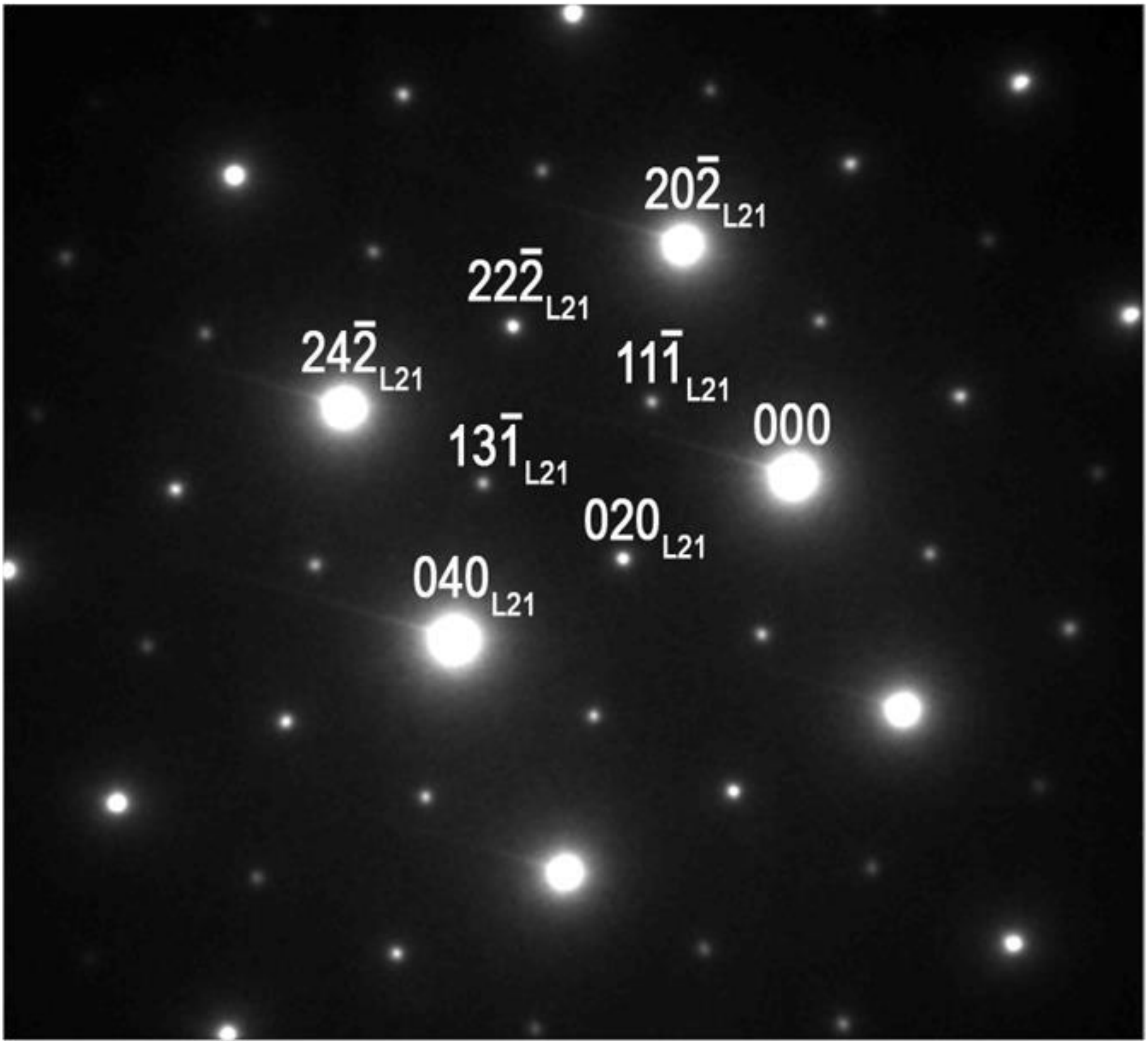

$20\bar{2}_{L21}$
$22\bar{2}_{L21}$
$24\bar{2}_{L21}$
$11\bar{1}_{L21}$
000
$13\bar{1}_{L21}$
020_{L21}
040_{L21}

Figure S1a

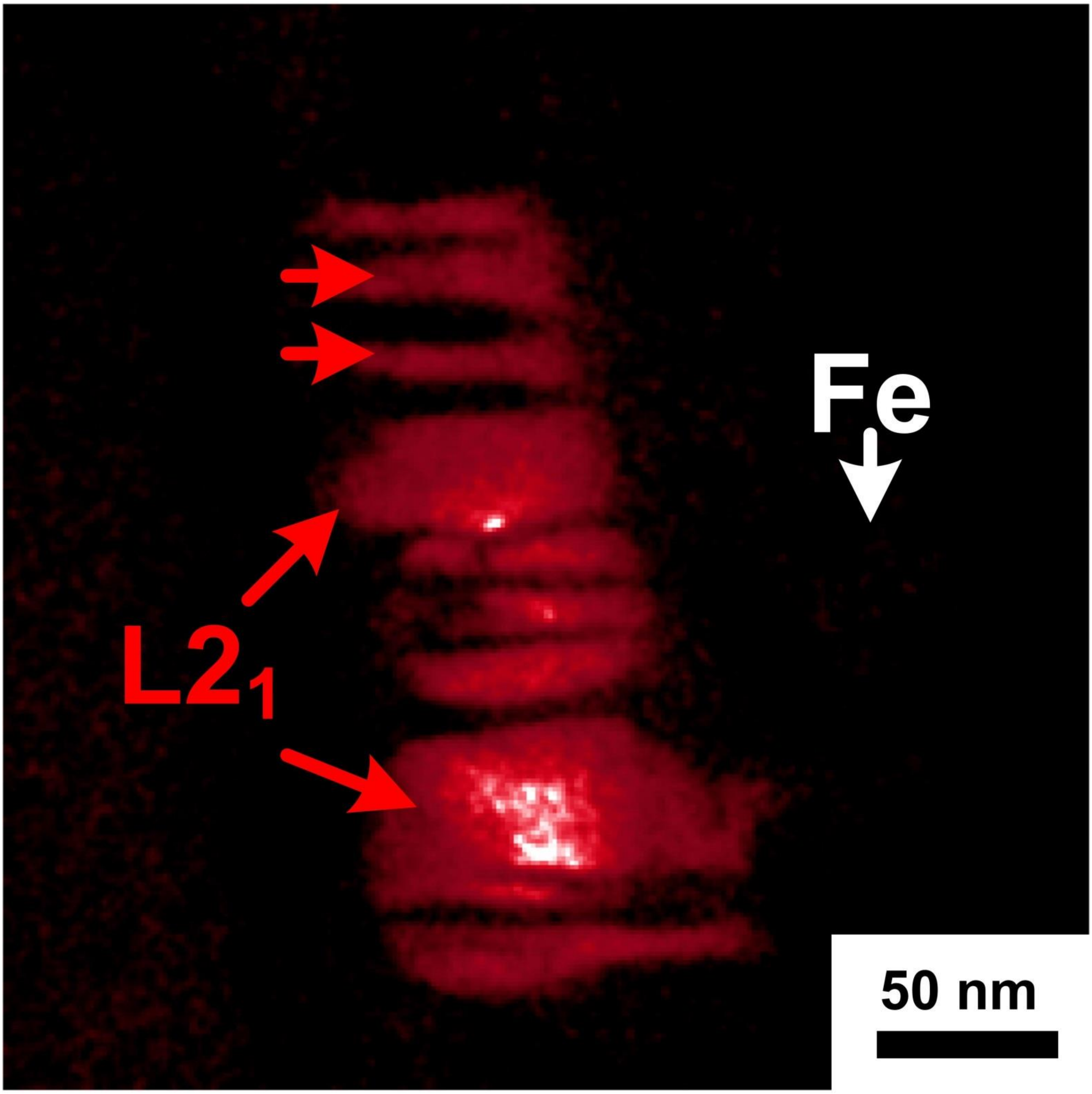

Figure S1b

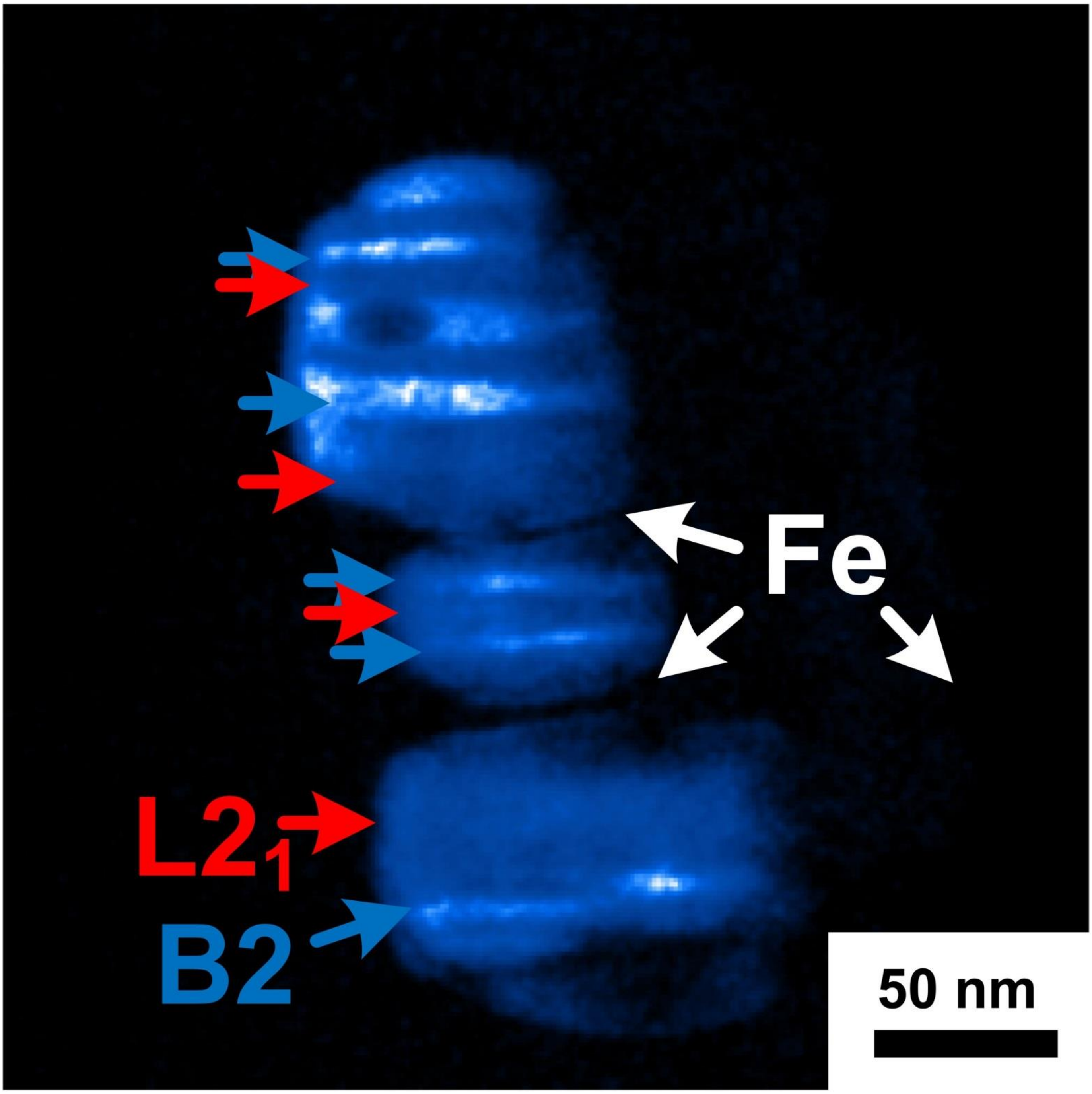

Figure S1c

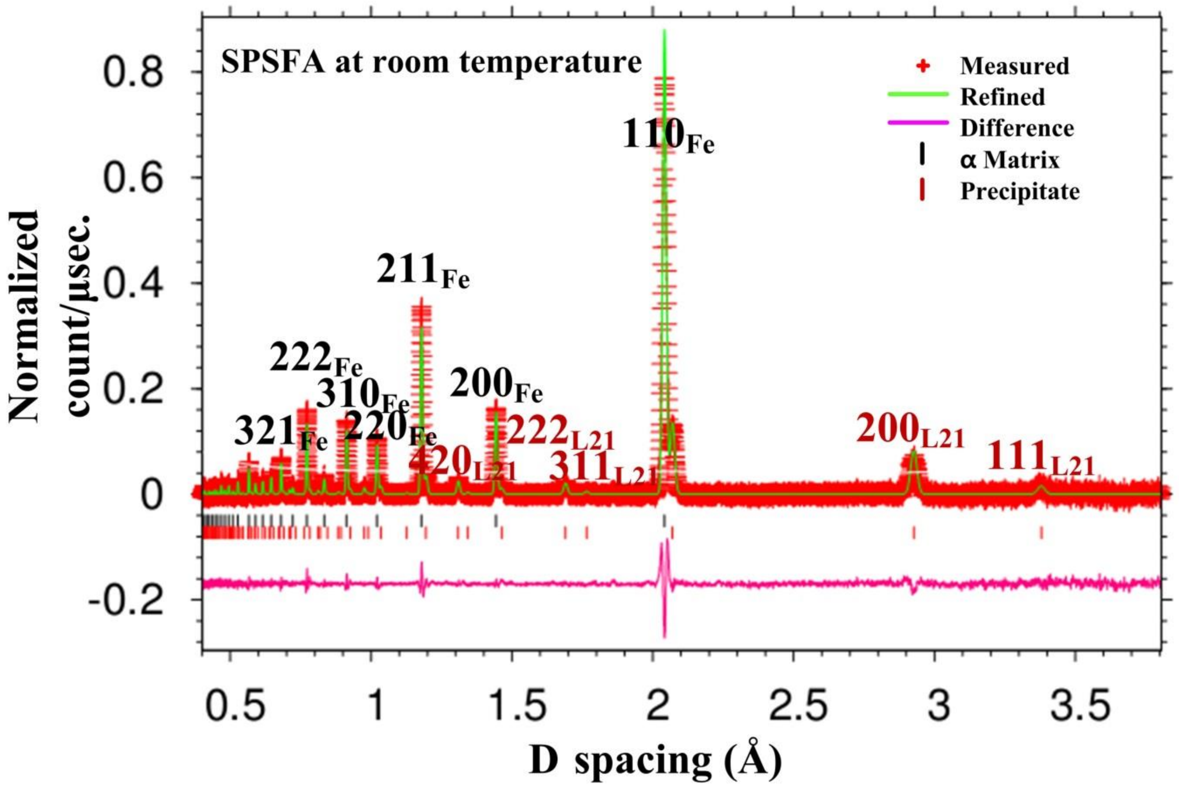
SPSFA at room temperature
Measured
Refined
Difference
α Matrix
Precipitate
110Fe
211Fe
222Fe
310Fe
200Fe
321Fe
220Fe
222L21
420L21
311L21
200L21
111L21
Normalized count/µsec.
0.8
0.6
0.4
0.2
0
-0.2
0.5
1
1.5
2
2.5
3
3.5
D spacing (Å)

Figure S2a

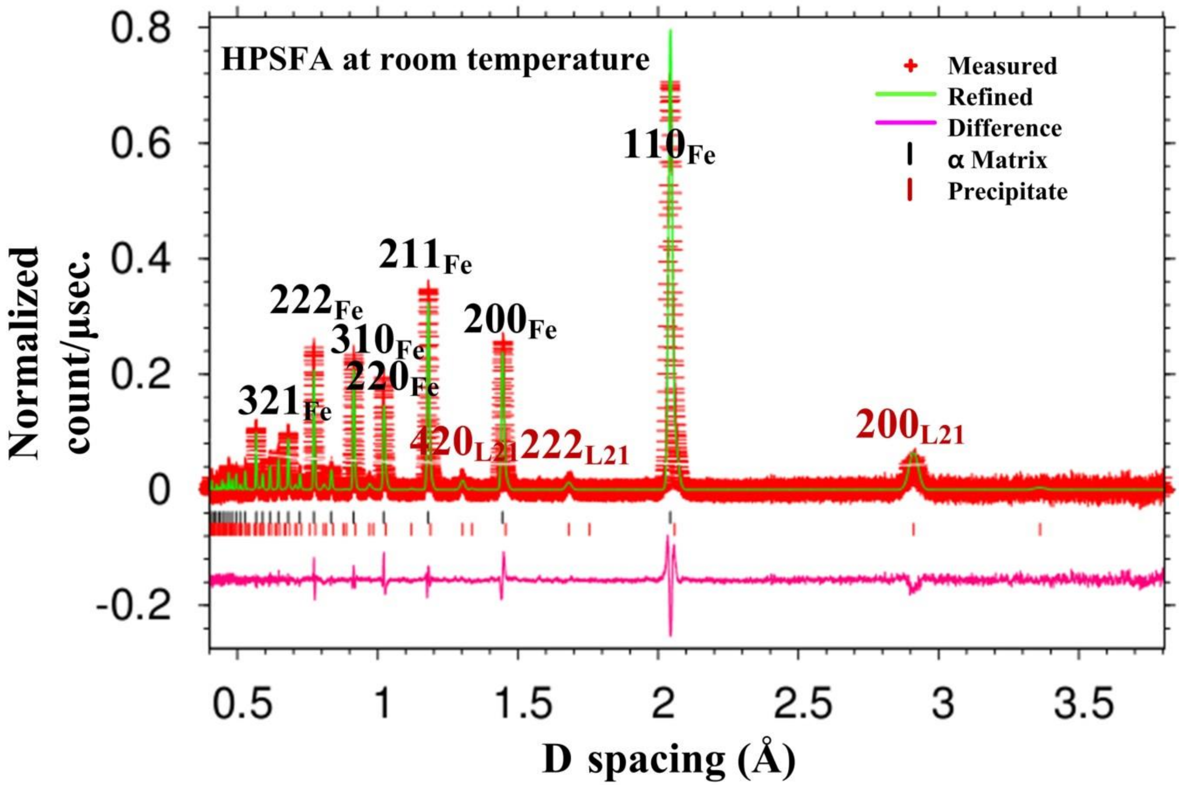
HPSFA at room temperature
Measured
Refined
Difference
α Matrix
Precipitate
110Fe
211Fe
222Fe
310Fe
220Fe
200Fe
321Fe
420L21
222L21
200L21
Normalized count/µsec.
D spacing (Å)
0.8
0.6
0.4
0.2
0
-0.2
0.5
1
1.5
2
2.5
3
3.5

Figure S2b

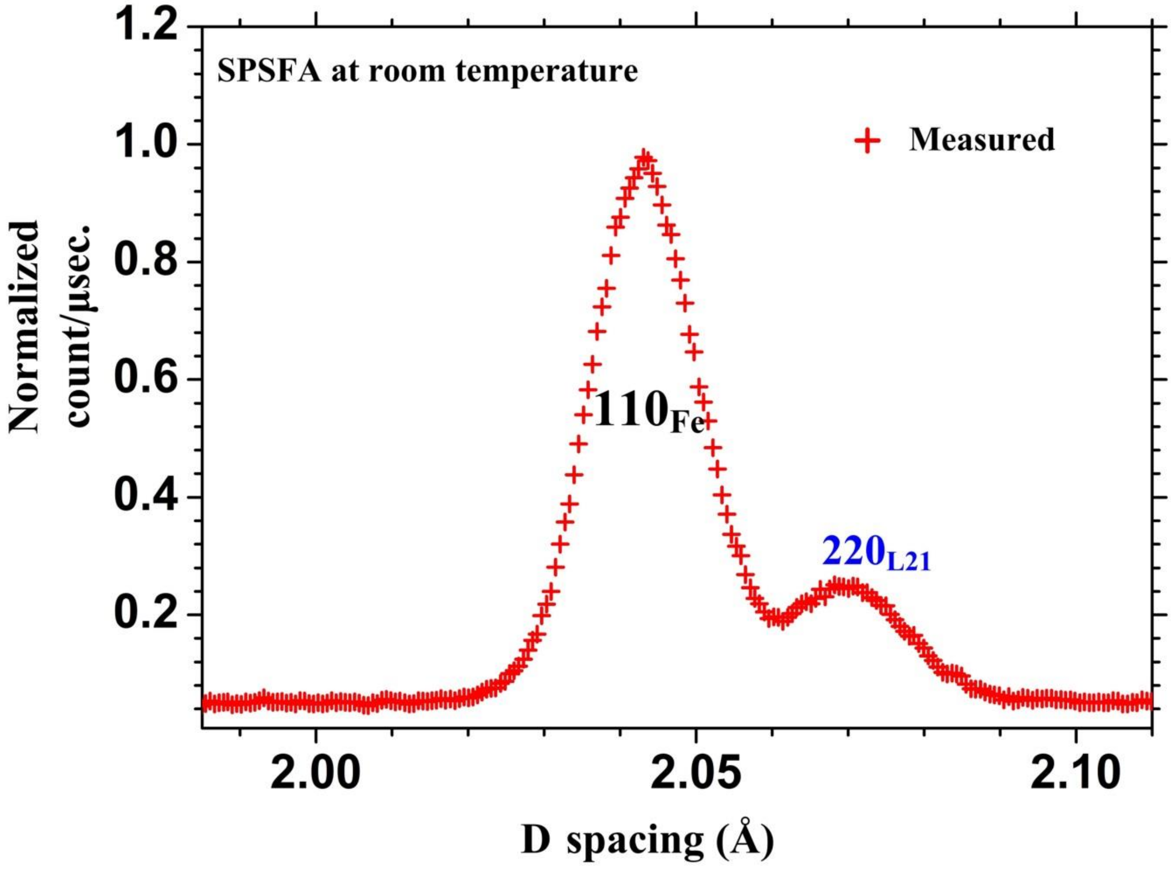

SPSFA at room temperature
Measured
110Fe
220L21
Normalized count/µsec.
D spacing (Å)
1.2
1.0
0.8
0.6
0.4
0.2
2.00
2.05
2.10

Figure S2c

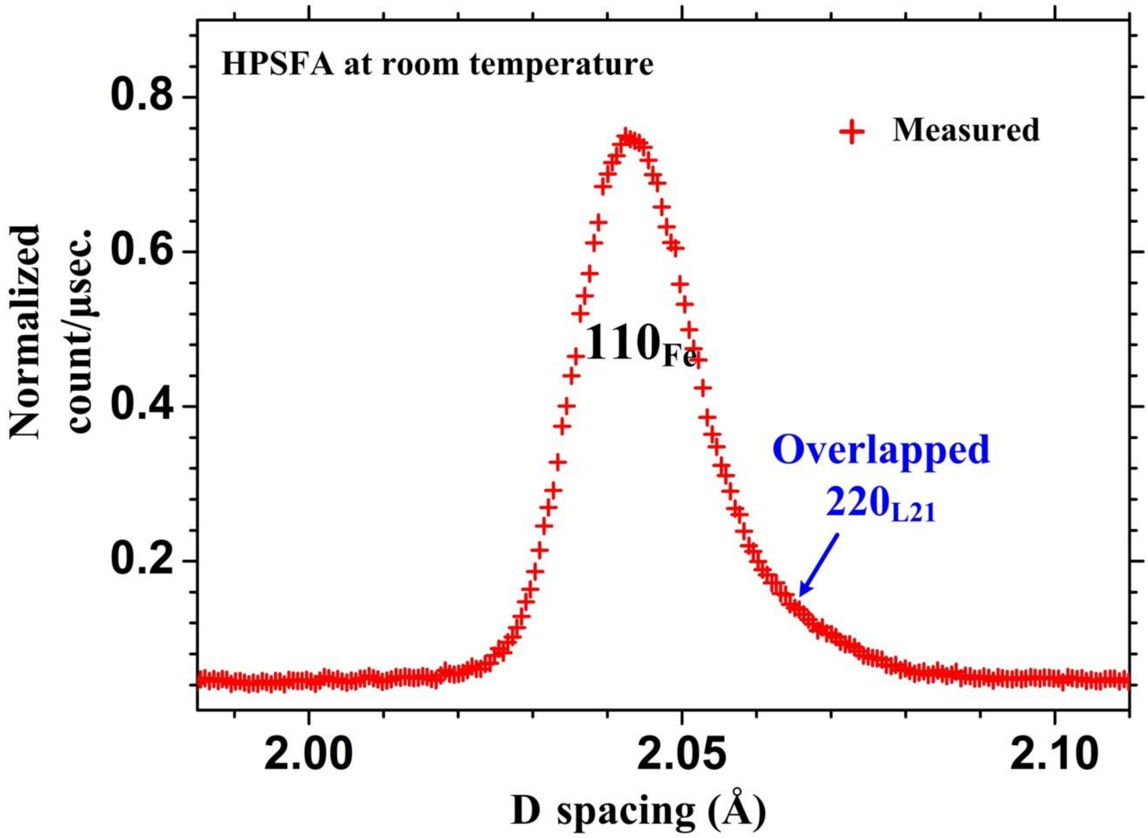
HPSFA at room temperature
Measured
110Fe
Overlapped
220L21
Normalized
count/µsec.
0.8
0.6
0.4
0.2
2.00
2.05
2.10
D spacing (Å)

Figure S2d

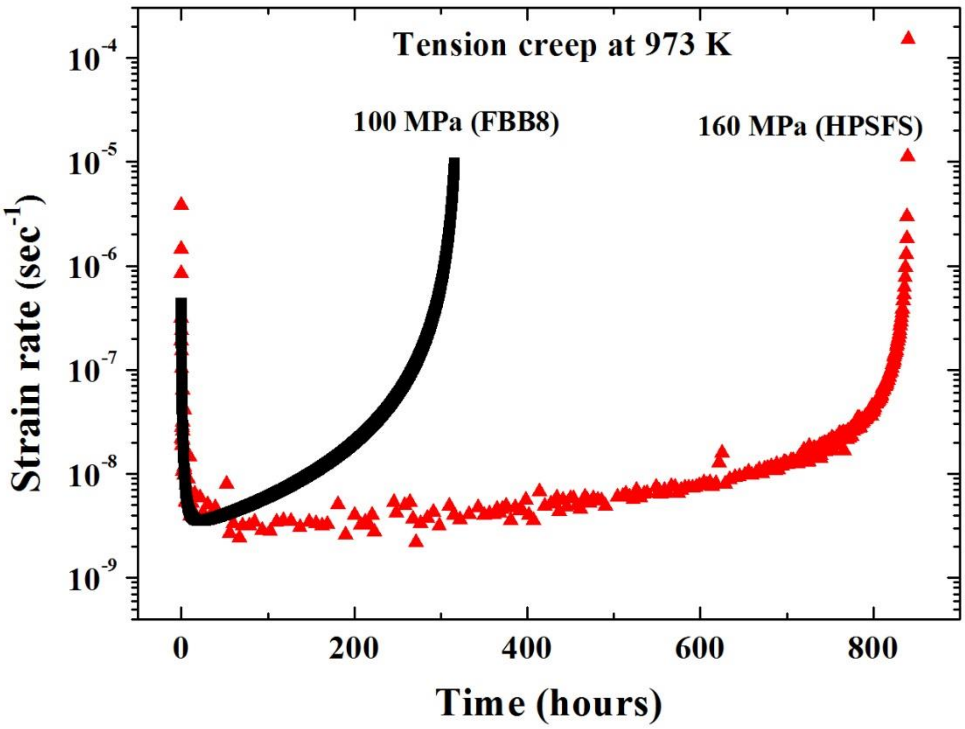
Tension creep at 973 K
100 MPa (FBB8)
160 MPa (HPSFS)
Strain rate (sec^{-1})
10^{-4}
10^{-5}
10^{-6}
10^{-7}
10^{-8}
10^{-9}
0
200
400
600
800
Time (hours)

Figure S3

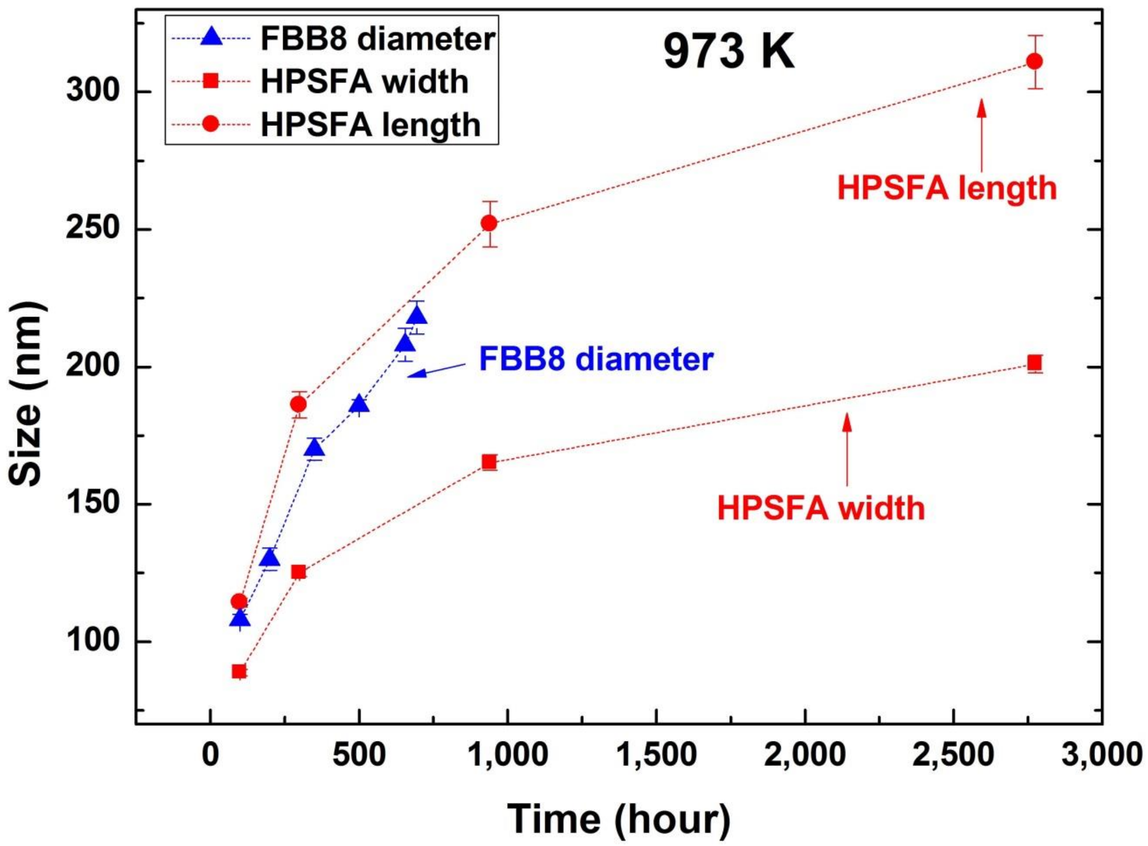

FBB8 diameter
HPSFA width
HPSFA length
973 K
HPSFA length
FBB8 diameter
HPSFA width
Size (nm)
300
250
200
150
100
0
500
1,000
1,500
2,000
2,500
3,000
Time (hour)

Figure S4

Table S1

Steel	C	Si	Mn	P	S	Ni	Cr	Cu	Mo	W	V	Nb	N	Al	B	Co
P92	0.10	0.23	0.61	0.008	0.001	0.21	9.09	-	0.43	1.83	0.20	0.064	0.046	0.003	0.0012	-
P122	0.13	0.27	0.61	0.014	0.001	0.34	10.15	0.49	0.36	1.94	0.20	0.055	0.057	0.017	0.0019	-
T91	0.09	0.29	0.35	-	-	0.28	8.70	0.032	0.90	-	0.22	0.072	0.044	0.001	-	-
T122	0.13	0.31	0.60	-	-	0.36	10.65	0.86	0.33	1.87	0.19	0.05	0.057	0.007	0.0024	-
12Cr	0.09	0.29	0.35	-	-	0.33	12.10	0.82	0.34	1.82	0.19	0.06	0.066	0.016	0.0030	-
0.002C	0.002	-	-	-	-	0.05	9.0	-	-	3.0	0.2	0.06	-	-	-	3.0

Table S2

Parameter / Phase	C_{11} (MPa)	C_{12} (MPa)	C_{44} (MPa)	n	q	h_0 (MPa)	τ_s (MPa)	τ_0 (MPa)	α (/°c)
**Fe**	113,800	96,000	51,100	10	1.0	100	157	120	1.66 e-6
**NiAl**	166,700	124,900	111,500	10	1.0	100	1,300	1,000	3.56 e-6
**Ni_2TiAl**	152,000	103,900	94,000	10	1.0	10	1,400	1,100	3.79 e-6
